# Subacute herpes simplex virus type 1 encephalitis as an initial presentation of chronic lymphocytic leukemia and multiple sclerosis: a case report

**DOI:** 10.1186/1752-1947-5-59

**Published:** 2011-02-11

**Authors:** Rashi L Singhal, Lourdes C Corman

**Affiliations:** 1 The University of Alabama at Birmingham, Huntsville, Alabama, USA

## Abstract

**Introduction:**

Herpes simplex virus type 1 encephalitis presents acutely in patients who are immunocompetent. We report what we believe to be the first published case of a subacute course of herpes simplex virus type 1 encephalitis in a patient with asymptomatic chronic lymphocytic leukemia who subsequently developed multiple sclerosis.

**Case presentation:**

A 49-year-old Caucasian woman with a history of fever blisters presented to the emergency department with a history of left temporal headache for four weeks, and numbness of the left face and leg for two weeks. A complete blood count revealed white blood cell count of 11,820 cells/mL, with an absolute lymphocyte count of 7304 cells/mL. The cerebrospinal fluid contained 6 white blood cells/μL, 63 red blood cells/μL, 54 mg glucose/dL, and 49 mg total protein/dL. Magnetic resonance imaging of the brain revealed meningoencephalitis and bilateral ventriculitis. Cerebrospinal fluid polymerase chain reaction for herpes simplex virus type 1 was positive, and the patient's symptoms resolved after ten days of treatment with parenteral aciclovir. Incidental findings on peripheral blood smear and flow cytometry testing confirmed chronic lymphocytic leukemia. One month later, she developed bilateral numbness of the hands and feet; a repeat cerebrospinal fluid polymerase chain reaction for herpes simplex virus type 1 at this time was negative. A repeat magnetic resonance imaging scan showed an expansion of the peri-ventricular lesions, and the cerebrospinal fluid contained elevated oligoclonal bands and myelin basic protein. A brain biopsy revealed gliosis consistent with multiple sclerosis, and the patient responded to treatment with high-dose parenteral steroids.

**Conclusion:**

Herpes simplex virus type 1 encephalitis is a rare presentation of chronic lymphocytic leukemia. Our patient had an atypical, subacute course, presumably due to immunosuppression from chronic lymphocytic leukemia. This unusual case of herpes simplex virus type 1 encephalitis emphasizes the importance of T cell function in diseases of immune dysregulation and autoimmunity such as chronic lymphocytic leukemia and multiple sclerosis. It raises the question of whether atypical presentations of herpes simplex virus encephalitis warrant deliberations on immunocompetence. The development of multiple sclerosis in our patient so soon after she received treatment for herpes simplex virus type 1 encephalitis raises the possibility that herpes simplex virus type 1 encephalitis in an immunosuppressed patient may trigger multiple sclerosis.

## Introduction

Herpes simplex virus type 1 (HSV-1) encephalitis is the most common cause of adult encephalitis worldwide. It classically occurs in patients under the age of 20 years due to primary infection, or in patients over the age of 50 years due to reactivation of latent infection. It is thought to occur sporadically in patients who are immunocompetent at the same rate as it does in patients who are immunocompromised [1].

HSV-1 encephalitis usually presents acutely, with general and focal signs of cerebral dysfunction such as fever, headache, altered mental status, behavioral changes, confusion, seizures, focal neurological findings, and abnormal cerebrospinal fluid (CSF) findings. The CSF of patients with HSV-1 encephalitis typically demonstrates a lymphocytic pleocytosis (white blood cells (WBC): 10 to 500 cells/μL), and erythrocytosis (red blood cells (RBC): 10 to 500 cells/μL). Levels of protein may be elevated to 60 to 700 mg/dL, and levels of glucose may be normal or slightly decreased (30 to 40 mg/dL).

Imaging of the brain with magnetic resonance imaging (MRI) classically demonstrates high signal intensity of the temporal lobe. Electroencephalography (EEG) results may show focal temporal abnormalities, such as spikes and slow waves or periodic sharp wave patterns. A diagnosis of HSV encephalitis is confirmed with identification of HSV in the CSF via polymerase chain reaction (PCR) testing or, less commonly, with identification of HSV in brain tissue via biopsy.

It is well established that patients with defects in cell-mediated immunity are at increased risk of severe oral or genital HSV infection; however, an increased frequency of HSV meningoencephalitis has not been reported. We report a subacute course of HSV-1 meningoencephalitis in a patient with undiagnosed chronic lymphocytic leukemia (CLL), who presented with biopsy-proven multiple sclerosis (MS) shortly after receiving treatment for HSV-1 encephalitis.

## Case presentation

A 49-year-old Caucasian woman with a history of migraines and herpes labialis presented to the emergency department (ED) with headache, and numbness and tingling in the left side of her face and her left leg. She related a history of recurrent sinusitis related to seasonal allergies, but with no recent nasal or pulmonary symptoms. She had developed peri-oral fever blisters, a low-grade fever, and a disabling left temporal headache four weeks earlier. Her headache was unlike the typical migraines that she periodically experienced, which usually responded to a combination of acetaminophen, aspirin, and caffeine. In addition, she experienced nausea, vomiting, and decreased appetite in association with her headaches that were accompanied by a 7.7 kg weight loss. She also exhibited personality changes and short-term memory loss. Her blisters healed in two weeks, but her headache and fatigue persisted. She subsequently developed numbness and subjective weakness in the left side of her face and her left leg one week prior to presentation. She sought care from her primary care provider, who diagnosed her with Bell's palsy and prescribed metoclopramide for her nausea. She was also referred to a neurologist who began treatment with aspirin for suspected transient ischemic attack. An MRI of the brain was pending at the time of her presentation to the ED.

**Figure 1:**
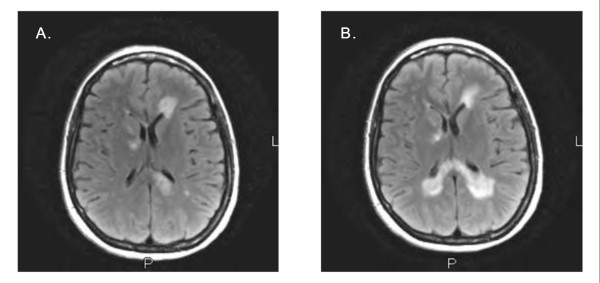
**Comparison of brain MRIs between patient's first (A) and second (B) admission.** A) Axial T2-flair MRI from 2 February 2009 demonstrating foci of peri-ventricular hyperintensity (more evident on the left than the right). Other findings included left greater than right lateral ventriculitis, basal meningitis, encephalitis involving the peri-ventricular and right basal ganglia white matter, and enhancing lesions in the left pons and the lower third of the medulla. B) Axial T2-flair MRI from 9 March 2009 demonstrating enlargement of prior hyperintense foci and new hyperintensity in the right posterior peri-ventricular white matter. Other findings included new enhancing lesions in the splenium of the corpus callosum, decreased enhancement in the right corona radiata and left frontal horn, and unchanged enhancements in the pons and medulla.

On admission, the patient's temperature was 37.9°C, blood pressure 166/96 mmHg, pulse 89 beats/minute, respiration rate 18 breaths/minute, and oxygen saturation 97.5% on room air. Her mental status and affect were normal. The distribution of dysesthesias was confirmed to be in the V2/V3 and L4/L5 dermatomes on physical examination. No motor deficits, other neurologic abnormalities, or lymphadenopathy were noted. A basic metabolic panel was remarkable for 3.2 mEq potassium/L (normal range 3.5 to 5.0). A complete blood count revealed 11.82 × 10^9 ^WBC/L, with 7.304 × 10^3 ^absolute lymphocytes/μL. Her CSF was clear and contained 6 WBCs/μL (89% lymphocytes), 63 RBCs/μL, 54 mg glucose/dL, and 49 mg total protein/dL; cell counts were taken from the fourth tube of CSF collected. Results of a chest radiograph were normal, and an MRI scan of the brain showed left greater than right ventriculitis, basal meningitis, and encephalitis of the peri-ventricular and right basal ganglia white matter (see Figure 1).

Treatment with intravenous dexamethasone, ceftriaxone, and aciclovir was initiated and the low potassium was replaced. The patient's headache and dysesthesias rapidly improved. On day 2, the hospital's pathology lab reported small, mature-appearing lymphocytes and smudge cells in her peripheral blood smear. Further immunological investigation revealed 259 absolute CD4+ T cells (normal range 400 to 1500), 389 absolute CD8+ T cells (normal range 275 to 780), and a CD4/CD8 ratio of 0.7 (normal range 0.9 to 3.7). Results of a CSF polymerase chain reaction (PCR) test for HSV-1 were positive, and results of serum HIV-1 and 2 antibody tests were negative. Ceftriaxone and dexamethasone were subsequently discontinued. The serum and CSF studies performed to rule out other infectious and vasculitic processes are listed in Table 1.

**Table 1:** Results of infectious and vasculitic and neoplastic studies performed on patient's first admission

Serum studies	Result (normal range or value)	CSF studies	Result (normal range)
*Ehrlichia *Ab titers			
*Anaplasma phagocytophilum *IgG	<1:64 (<1:64)	Aerobic/anaerobic culture with Gram stain	No organisms seen, no growth at 3 days
*Ehrlichia chaffeensis *IgG	<1:64 (<1:64)		

*Bartonella *Ab titers			
*Bartonella henselae *IgG and IgM	<1:128 and <1:20 (<1:128 and <1:20)	Fungal preparation and culture	No fungal elements seen, no fungus recovered at 4 weeks
*Bartonella quintana *IgG and IgM	<1:128 and <1:20 (<1:128 and <1:20)		

Lyme disease antigen PCR	Negative	AFB culture with smear	No AFB seen, no AFB recovered at 8 weeks.

RPR	Non-reactive	VDRL	Negative

Viral hepatitis panel			
HA Ab (IgM)	Non-reactive		
HBcAb (IgM) and HBsAg	Non-reactive	CMV PCR	Negative
HC Ab	Non-reactive		

LCMV IgG and IgM Ab titer	<1:16 and <1:20 (<1:16 and <1:20)	LCMV IgG and IgM Ab titer	<1:1 and <1:1 (<1:1 and <1:1)

WNV IgG and IgM Ab	Negative	WNV IgG and IgM Ab	Negative

Toxoplasma IgG and IgM Ab titer	36 IU/mL and <0.55 (<4 and <0.55)	WNV PCR	Negative

Cryptococcal Ag	Negative	Enterovirus PCR	Negative

ESR (mm/hour)	9 (0 to 20)	VZV PCR	Negative

CRP (mg/dL)	0.2 (<0.5)	ACE (ACE units)	<4 (<4)

Expanded ANA screen: Abs to dsDNA, chromatin, Sm, RNP, Sm-RNP, Ribosomal protein, SS-A, SS-B, Jo-1, Scl-70, and Centromere B	Negative		

Serum protein electrophoresis	No monoclonal bands identified		

Ab = antibody; ACE = angiotensin-converting enzyme; AFB = acid-fast bacilli; Ag = antigen; ANA = anti-nuclear antibody; CMV = cytomegalovirus; CRP = C-reactive protein; dsDNA = double-stranded DNA; ESR = erythrocyte sedimentation rate; HA = hepatitis A; HBcAb = anti-hepatitis B core antibody; HBsAg = hepatitis B surface antigen; LCMV = lymphocytic choriomeningitis virus; PCR = polymerase chain reaction; RNP = ribonucleoprotein; RPR = rapid plasma reagin; SS-A/B = Sjögren's syndrome antigen A/B; VDRL = Venereal Disease Research Laboratory; VZV = varicella zoster virus; WNV = West Nile virus.

A diagnosis of CLL was confirmed by the expression of CD5, CD19, and CD20 antigens on monoclonal B cells with κ light chain restriction on blood flow cytometry. Fluorescent *in situ *hybridization later revealed a 13 q deletion, the most common cytogenetic abnormality seen among patients with CLL. The patient's γ-globulin level was 839 mg/dL (normal range 700 to 1600). After eight days on intravenous aciclovir, her symptoms had completely resolved and she was discharged on oral valaciclovir. She remained well for the next few weeks.

**Figure 2:**
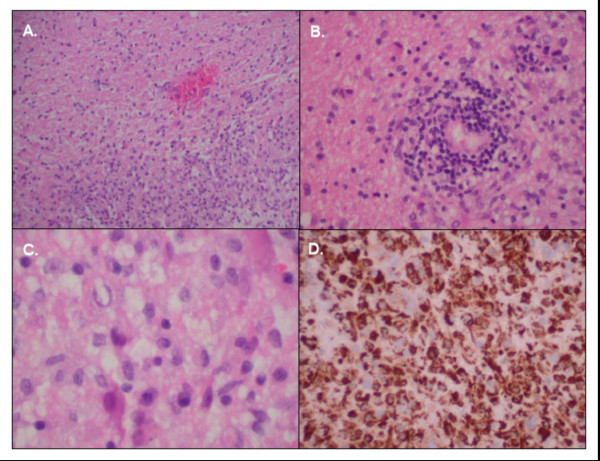
**Left occipital brain biopsy performed on 15 March 2009.** A) Hematoxylin and eosin (H&E) stain (100×) showing a sharp border between relatively normal neuropil inferiorly and a paler area of gliosis representing a lack of myelin superiorly. B) H&E stain (200×) showing a peri-vascular cuff consisting of lymphocytes and histiocytes. C) H&E stain (400×) showing an inflammatory infiltrate rich with histiocytes. D) Immunoperoxidase stain (200×) for CD68 macrophages showing strong positivity in areas of gliosis. The final pathological diagnosis was gliosis and reactive changes consistent with demyelinating disease. The findings were negative for lymphoma. An immunostain for herpes simplex virus (HSV) was also negative. Other immunohistochemistry stains were as follows: B cell marker CD20 was positive in a few B cells, T cell marker CD3 was positive for several T cells, B cell marker CD79 was positive in a few B cells, and proliferation marker Ki-67 was low at 3%.

Then, one month after discharge, she developed new bilateral numbness of the hands and feet and was found to have cervical lymphadenopathy on physical examination. A repeat MRI showed increased numbers and size of peri-ventricular lesions (see Figure 1), and she was readmitted. A repeat CSF PCR test for HSV-1 was negative, but 10 oligoclonal bands and a myelin basic protein level of 5.7 ng/mL (normal value <1.5) were found. Table 2 details other laboratory test results for comparison between the patient's first and second admissions. Computed tomography (CT) scans revealed extensive cervical lymphadenopathy, slightly enlarged axillary nodes bilaterally, and early lymphadenopathy among the mesentery. A brain biopsy was performed to rule out CNS lymphoma, and it demonstrated gliosis consistent with MS with no evidence of lymphoma (see Figure 2). She received high-dose parenteral steroids for five days, with symptom resolution occurring within the first two to three days. She was discharged on oral prednisone treatment, to follow up with her neurologist and oncologist. Six months later, the patient was still asymptomatic, having started interferon-β therapy for MS and not yet needing treatment for Rai stage zero CLL.

**Table 2:** Comparison of laboratory studies performed during patient's first and second admissions

Laboratory studies	First admission (2 to 9 February)	Second admission (11 to 21 March)	Normal range or value
Complete blood count			
WBC (× 10^9^/L)	11.82	9.12	4.8 to 10.8
Absolute lymphocytes (× 10^3^/μL)	7.29	5.84	1.20 to 3.40

CSF lumbar puncture			
RBC (cells/μL)	63	6	
WBC (cells/μL)	6	8	0 to 5
Neutrophils (%)	0	3	
Lymphocytes (%)	83	86	
Monocytes (%)	17	10	
Number of cells counted	18	Not measured	
Glucose (mg/dL)	54	55	
Total protein (mg/dL)	49	40	15 to 45

CSF rapid PCR for HSV-1	Positive	Negative	

CSF cytology	Negative	Negative	

CSF flow cytometry	Negative	Negative	

Oligoclonal banding			
CSF bands	Not measured	10	<4
Serum bands		0	

CSF IgG index			
CSF IgG index		0.9	<0.85
CSF IgG (mg/dL)		6.11	<8.1
CSF albumin (mg/dL)		32.3	<27.0
CSF IgG/albumin	Not measured	0.19	<0.21
CSF synthesis rate (mg/24 hours)		14.24	<12
Serum IgG (mg/dL)		849	600 to 1500
Serum albumin (mg/dL)		4010	3200 to 4800
Serum IgG/albumin		0.2	<0.4

CSF myelin basic protein (ng/mL)	Not measured	5.7	<1.5

This table demonstrates the available studies performed during each of the patient's admissions that pertain to her diagnoses of chronic lymphocytic leukemia (complete blood count, CSF cytology, CSF flow cytometry), Herpes simplex virus type-1 encephalitis (CSF lumbar puncture, CSF rapid PCR for HSV-1), and multiple sclerosis (oligoclonal banding, CSF IgG index, CSF myelin basic protein).

CSF = cerebrospinal fluid; HSV = herpes simplex virus; PCR = polymerase chain reaction; RBC = red blood cells; WBC = white blood cells.

## Discussion

HSV-1 encephalitis usually presents fulminantly in immunocompetent individuals with fever, altered mental status, seizures, CSF and EEG findings, and temporal lobe lesions on MRI. Our patient's illness began with recurrent herpes simplex labialis, which spread to the left trigeminal nerve and left L5 nerve root over the course of four weeks. Besides her dysesthesias, her symptoms included headache, low-grade fever, weight loss, mild personality change and short-term memory loss, which were no longer exhibited on presentation. Her CSF contained mildly elevated levels of lymphocytes (WBC: 6 cells/μL), erythrocytes (RBC: 63 cells/μL), and protein (49 mg/dL); owing to the fact that cell counts were taken from the fourth tube collected, the probability of a traumatic lumbar puncture is low. Beyond her MRI findings of peri-ventriculitis and basal meningitis particularly over the brainstem, our patient had no temporal lobe abnormalities, although approximately 10% of patients with PCR-proven HSV encephalitis do not demonstrate temporal lobe involvement. In addition, other cases of brainstem encephalitis due to HSV have been reported with viral reactivation possibly occurring in the trigeminal nerve (relevant references are available upon request from the corresponding author). Regarding involvement of white matter, other cases of extratemporal HSV encephalitis have also shown this pattern on neuroimaging, particularly during chronic or subacute phases of illness; often being associated with clinical relapse and viral persistence; and sometimes showing demyelination in addition to edema, inflammatory change, and viral inclusions on a microscopic level (relevant references are available upon request from the corresponding author). Overall, our patient's clinical presentation, borderline pleocytosis in the CSF, and extratemporal findings on MRI were consistent with a more subacute course of HSV-1 encephalitis proven by PCR, presumably due to immunosuppression from CLL.

Patients with CLL often manifest hypogammaglobulinemia and neutropenia early in the course of the disease, with eventual T cell deficiency leading to recurrent sinopulmonary infections with Gram-negative bacteria, fungi, and herpes zoster and simplex viruses. However, HSV-1 encephalitis is a rare presentation of CLL. Other central nervous system (CNS) infections have been reported in patients with CLL, namely progressive multifocal leukoencephalopathy, West Nile virus encephalitis, and *Listeria monocytogenes *encephalitis. The occurrence of HSV-1 encephalitis in immunocompromised patients has rarely been reported. Classic clinical presentations with atypical laboratory studies have been described in a patient with metastatic colon cancer four weeks after receiving chemotherapy [2], three patients who were immunosuppressed with either Hodgkin's or non-Hodgkin's lymphoma [3-5], and another patient with glioblastoma multiforme on dexamethasone [5]. Atypical clinical presentations of HSV encephalitis have been described in patients receiving steroids, and also occur in 2% of patients infected with human immunodeficiency virus (HIV) with neurological symptoms, usually in association with CD4 T cell counts <200 cells/μL [6].

The literature exploring the association of MS with CLL is very limited. Neither the development of CLL prior to the development of MS, nor the reverse, have been shown to have an association in retrospective studies ([7]; additional references are available upon request from the corresponding author). Only one study has shown an increased risk of Hodgkin's and non-Hodgkin's lymphoma in first-degree relatives of patients with MS, and this is thought to be related to shared environmental and constitutional factors such as infection with Epstein-Barr virus or expression of the HLA-DR2 allele.

**Figure 3:**
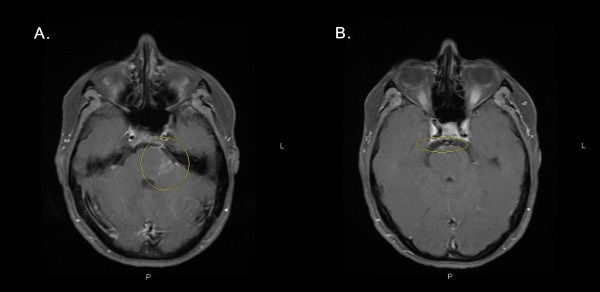
**Brain MRI demonstrating basal meningitis during the patient's first admission**. Axial T1-SE fat-suppression contrast-enhanced MRI scans showed abnormal signal intensity of the basal meninges, particularly involving the left cerebellar peduncle (A) and the right anterior mesencephalic-pontine junction (B).

The development of clinical symptoms and signs of MS in our patient so soon after her episode of HSV-1 encephalitis, as well as her earlier atypical peri-ventricular pattern of MRI findings, suggests a possible association between the infection and subsequent diagnosis of MS. On the one hand, it is possible that our patient's initial illness was an early manifestation of MS with asymptomatic HSV-1 colonization in the CNS, and that her initial improvement could be attributable to receiving dexamethasone for 1 day. Of note, detection of HSV DNA in CSF by PCR has a sensitivity of 98% and a specificity of >95% [8]. True positives have been reported in patients who are asymptomatic, and false positives have been reported due to cross-contamination. Additionally, the MRI from our patient's first admission demonstrated basal meningitis, which is an atypical feature of MS (see Figure 3). On the other hand, our patient's recent recurrence of herpes labialis, the positive CSF PCR for HSV-1, and her continued improvement over nine days while receiving intravenous aciclovir support the argument that HSV-1 infection was the reason for her initial illness. Regarding the nature of the illness, CSF PCR for HSV has been recently used by others to diagnose mild cases of HSV encephalitis, to diagnose atypical cases of adult HSV encephalitis involving polyradiculoneuritis and inflammatory syndrome with arachnoiditis, and to define a pathogenic role of HSV in episodes unlikely to be entirely CNS viral infections (relevant references are available upon request from the corresponding author). Furthermore, the fact that the patient's repeat CSF PCR result for HSV-1 was negative on her second admission helps support that her initial encephalitis was related to HSV-1 even if some demyelination due to MS had already begun. Although studies of the CSF such as oligoclonal banding were not performed or indicated during her first admission to explore the likelihood of MS, the patient denied any history of urinary incontinence, vision loss, unilateral blurred vision, double vision, gait disturbance, or paresthesias before her current illness. However, the expansion of previous areas of peri-ventriculitis on the patient's second admission do raise the possibility of her first admission involving concurrent HSV-1 encephalitis and MS.

A recent study of patients with MS has demonstrated alterations in dendritic cell antigens and interferon expression in response to HSV-1 challenge to mononuclear cells [9]. Whether such an impaired immune response to viral infection is present before the development of MS is unknown. Additionally, patients with MS are known to have elevated antibody titers to HSV-1 in the CSF, but this is purported to be non-specific and studies of CSF PCR for HSV-1 in patients with MS have failed to find a statistically significant association [10]. There is evidence of association between infection with Epstein-Barr virus (EBV) and subsequent diagnosis of MS, as well as between CNS infection with human herpesvirus 6 variant A (HHV6A) and concurrent MS [10]; however causal relationships are yet unproven. Our patient did not undergo serological EBV testing or CSF studies for HHV6 during her admissions.

## Conclusion

No definitive association can be inferred regarding the development of MS in our patient so soon after her diagnoses of CLL and HSV-1 encephalitis, but this occurrence warrants reporting. This atypical presentation of HSV-1 infection in a patient with undiagnosed CLL emphasizes the importance of T cell function in diseases of immune dysregulation and autoimmunity such as CLL and MS. It raises the issue of whether patients with atypical presentations of HSV encephalitis deserve a deliberation on their state of immunocompetence.

## Consent

Written informed consent was obtained from the patient for publication of this case report and any accompanying images. A copy of the written consent is available for review by the Editor-in-Chief of this journal.

## Competing interests

The authors declare that they have no competing interests.

## Authors' contributions

RLS and LCC followed the course of the patient's illness in the hospital. RLS collected, analyzed, and interpreted pertinent information from the literature and patient data including laboratory and imaging studies, biopsy results, and overall clinical progress. LCC reviewed the text and figures with RLS over several months, making suggestions for revisions and further literature review. All authors read and approved the final manuscript.
